# Heparanase and Autoimmune Diabetes

**DOI:** 10.3389/fimmu.2013.00471

**Published:** 2013-12-26

**Authors:** Charmaine J. Simeonovic, Andrew F. Ziolkowski, Zuopeng Wu, Fui Jiun Choong, Craig Freeman, Christopher R. Parish

**Affiliations:** ^1^Diabetes/Transplantation Immunobiology Laboratory, The John Curtin School of Medical Research, The Australian National University, Canberra, ACT, Australia; ^2^Cancer and Vascular Biology Group, Department of Immunology, The John Curtin School of Medical Research, The Australian National University, Canberra, ACT, Australia

**Keywords:** heparanase, heparan sulfate, islet, diabetes, inflammation, vascular complications

## Abstract

Heparanase (Hpse) is the only known mammalian endo-β-d-glucuronidase that degrades the glycosaminoglycan heparan sulfate (HS), found attached to the core proteins of heparan sulfate proteoglycans (HSPGs). Hpse plays a homeostatic role in regulating the turnover of cell-associated HS and also degrades extracellular HS in basement membranes (BMs) and the extracellular matrix (ECM), where HSPGs function as a barrier to cell migration. Secreted Hpse is harnessed by leukocytes to facilitate their migration from the blood to sites of inflammation. In the non-obese diabetic (NOD) model of autoimmune Type 1 diabetes (T1D), Hpse is also used by insulitis leukocytes to solubilize the islet BM to enable intra-islet entry of leukocytes and to degrade intracellular HS, an essential component for the survival of insulin-producing islet beta cells. Treatment of pre-diabetic adult NOD mice with the Hpse inhibitor PI-88 significantly reduced the incidence of T1D by ~50% and preserved islet HS. Hpse therefore acts as a novel immune effector mechanism in T1D. Our studies have identified T1D as a Hpse-dependent disease and Hpse inhibitors as novel therapeutics for preventing T1D progression and possibly the development of T1D vascular complications.

## Introduction

Heparanase (Hpse) is an endo-β-d-glucuronidase that degrades the glycosaminoglycan heparan sulfate (HS). Cloning studies have identified that catalytically active Hpse is encoded by a single mammalian gene (1–3). Hpse is initially produced as an inactive pre-proenzyme which undergoes post-translational processing to yield a 65 kDa proenzyme for secretion. Proteolytic cleavage of proheparanase by the cysteine protease cathepsin L leads to the formation of catalytically active Hpse, a heterodimer consisting of a 50 kDa (human) or 48 kDa (mouse) polypeptide non-covalently bound to a 8 kDa peptide (1, 2, 4–8). HS is a linear polysaccharide that consists of a repeating disaccharide composed of *N*-acetylated glucosamine (GlcNAc) and uronic acid [glucuronic acid (GlcA) or iduronic acid (IdoA)]. HS biosynthesis occurs in the Golgi compartment of cells, with the assembly of component sugar residues occurring directly onto the core proteins of heparan sulfate proteoglycans (HSPGs) (9–11). During the polymerization of HS chains, selected sugar residues are chemically modified by a suite of enzymes (*N*-deacetylase-*N*-sulfotranferase, C5 epimerase, and 2, 3, and 6-*O*-sulfotransferases), resulting in HS chains with regions that are highly sulfated and other regions of lower or no sulfation (10, 11). The sulfated regions of HS, in particular, bind to a vast array of bioactive ligands that include cytokines, chemokines, growth factors, adhesion molecules, lipases, and proteases (12, 13).

Typically, HSPGs are localized at the cell surface (e.g., syndecans 1–4, glypicans 1–6), in the extracellular matrix (ECM), in basement membranes (BMs) (e.g., perlecan, collagen type XVIII, and agrin) and have been identified in the nucleus of certain cells (14, 15). Secreted proheparanase rapidly interacts with cell surface HSPGs and the proheparanase-HSPG complex subsequently undergoes endocytosis. Similarly, Hpse can be internalized after binding to cell surface lipoprotein receptor-related proteins (LPRs) and mannose-6-phosphate receptors (MPRs) (16). Internalized proheparanase is cleaved by intracellular cathepsin L at acidic pH in late endosomes or lysosomes, to form catalytically active Hpse which can either degrade co-endocytosed HS, thereby regulating the turnover of cell-associated HS, or undergo storage within the lysosomes for subsequent secretion (6, 17–21). Optimal Hpse-mediated cleavage of glycosidic bonds in HS occurs at pH 5.5–6.0 and typically at sites adjacent to *N*- or 6-*O*-sulfated glucosamine (16, 22), e.g., the linkage of glucuronic acid to 6-*O*-sulfated glucosamine (23). HS in BMs and ECM is degraded by Hpse secreted by platelets, endothelial cells, leukocytes, and metastasizing tumor cells (12). In these settings, Hpse activity can result from (i) activation of proheparanase bound to cell surface HSPG or to cation-independent MPRs (CIMPRs) by an extracellular source of cathepsin L, e.g., produced by macrophages (24, 25); (ii) cytokine-, fatty acid-, or nucleotide-stimulated release of an intracellular pool of catalytically active Hpse (26–29) which may be subsequently captured by cell surface receptors such as CIMPRs (25); or (iii) platelet degranulation (30). This regulated release of Hpse in the local microenvironment limits the availability of Hpse activity, preserving the essential and diverse biological functions of HS.

Heparanase also exhibits non-enzymatic functions which impact on cell signaling, adhesion, and migration, as well as on gene expression. Such functions are generally expressed at neutral pH (31–33). Interaction of Hpse with cell surface receptors on endothelial cells activates intracellular Akt, PI3K, and p38 kinase signaling to stimulate cell migration and Src kinase-mediated upregulation of vascular endothelial growth factor (VEGF) for angiogenesis (6, 18, 34). Hpse lacking catalytic enzyme activity has been shown to increase the expression of certain growth factors (35) and to facilitate cell binding to HS in the ECM and to endothelial cells *in vitro* (32).

Intra-nuclear Hpse modulates intra-nuclear HS/HSPGs and exerts direct effects on gene transcription. Transfer of Hpse to the nucleus occurs via Hsp90 in endothelial cells following fatty acid stimulation (29). Intra-nuclear Hpse decreases the level of the HSPG syndecan-1 in the nucleus of myeloma cells (14) and cleaves nuclear HS which in turn inhibits histone acetyltransferases (36). Recently, active Hpse has been reported to directly mediate epigenetic effects by regulating histone methylation, a process that directly influences the transcription of certain immune response genes involved in T-cell migration and function, e.g., IL-2 and IFN-γ (37). Hpse was also found to bind to the promoters of micro-RNAs involved in T-cell differentiation (37) and to influence the transcription of genes encoding enzymes involved in glucose metabolism (29). Such nuclear roles for Hpse, either with or without HS-degrading activity, would be expected to impact on T cells in inflammatory responses.

## Heparanase and Inflammation

Heparan sulfate has several important biological functions which are regulated by Hpse in inflammation. An inflammatory response is generated when leukocytes are rapidly recruited from the blood to sites of tissue injury. In the early stages of inflammation, cell surface HS on cytokine-activated or inflamed endothelial cells functions in presenting lymphocyte-attractant chemokines to leukocytes in the vascular lumen (12, 38). The subsequent immobilization of the leukocytes (e.g., T cells) at the endothelial cell surface is enhanced by the binding of chemokine-activated integrins on the leukocytes to adhesion molecules such as ICAM-1 or VCAM-1 expressed on endothelial cells. Such interactions could potentially be facilitated by the binding of T cell-bound inactive Hpse to HS expressed on the surface of endothelial cells (12, 32, 33). The chemokine-binding role for endothelial cell surface HS may also function in establishing a chemokine gradient to *direct* leukocyte migration across the endothelium (12). Having crossed the blood vessel wall, most probably by passing between endothelial cells, inflammatory leukocytes employ degradative mechanisms to traverse the sub-endothelial BM. In fact BM HS, particularly associated with the HSPG perlecan, helps the BM to act as a barrier to leukocyte migration. This barrier property is attributed to the length of HS chains (up to 400 sugar residues) and to their intrinsic capacity to interact with other BM matrix proteins, forming a cell-impenetrable scaffold (12). To overcome this obstacle, leukocytes including T cells (39, 40), nearby endothelial cells (26) and possibly platelets (40) produce Hpse to degrade BM HS and proteases to destroy BM matrix proteins. The disassembly of the BM matrix components aids the passage of leukocytes across the BM and their entry into the surrounding tissue. Similarly, Hpse is released by inflammatory leukocytes to solubilize HS in the ECM of underlying tissues and to aid their navigation to sites of inflammation (12). During the course of the degradation of extracellular HS, HS-bound cytokines and chemokines can be liberated into the local microenvironment, potentially augmenting cell recruitment and exacerbating the inflammatory response (12).

The role for Hpse as a “path-maker” required by migrating leukocytes is of particular significance for T cell-mediated autoimmune diseases. Indeed, Hpse activity represents a prime target for anti-inflammatory drug development. Experimental autoimmune encephalitis (EAE; an experimental model of multiple sclerosis) in rats was largely prevented by *in vivo* treatment with sulfated polysaccharides. This effect was attributed to the inhibition of Hpse produced by activated T cells, which in turn blocked the solubilization of the sub-endothelial BM (41, 42). In a delayed-type hypersensitivity (DTH) experimental model of inflammation, inhibition of endothelial cell-derived Hpse prevented the degradation of sub-endothelial BM HS and leukocyte migration (27). Hpse, possibly produced by inflammatory cells in rheumatoid arthritis in humans, may release cytokines and/or chemokines from degraded HS in the ECM of rheumatoid joints, promoting joint destruction (43). In ulcerative colitis and Crohn’s disease, which represent chronic inflammatory disorders, Hpse is preferentially produced by inflamed gut epithelial cells to drive a local circuit of inflammation (24, 44). A role for Hpse has therefore been established in a broad range of inflammatory conditions.

## Heparanase and the Pathogenesis of Type 1 Diabetes

Type 1 diabetes is an autoimmune disease which has been extensively studied in non-obese diabetic (NOD) mice, a recognized preclinical model of T1D in humans. During T1D, the insulin-producing beta cells in the islets of Langerhans in the pancreas are selectively destroyed by a T cell-mediated autoimmune response (45). The priming of autoreactive T cells to their cognate beta cell-specific autoantigens most probably occurs in the draining pancreatic lymph nodes, possibly as a consequence of both the abnormal responsiveness of effector T cells and inadequate control by regulatory T cells (46). Histological studies of NOD female mice at an early age (~6–7 weeks) revealed that leukocytes initially accumulate around the periphery of the islets, forming foci of non-destructive inflammation (insulitis). In adult pre-diabetic mice, the insulitis advances to a destructive phenotype, with peri-islet inflammatory leukocytes invading the islet cell mass (45). However, this pathology does not occur as a synchronized process throughout the pancreas, and the proportion of affected islets as well as the severity of leukocyte invasion progressively increases with time. Clinical symptoms of T1D are observed in 60–80% of female NOD mice from ~100 days of age or older, and are characterized by blood glucose levels exceeding >20 mmol/L (hyperglycemia).

**Figure 1:**
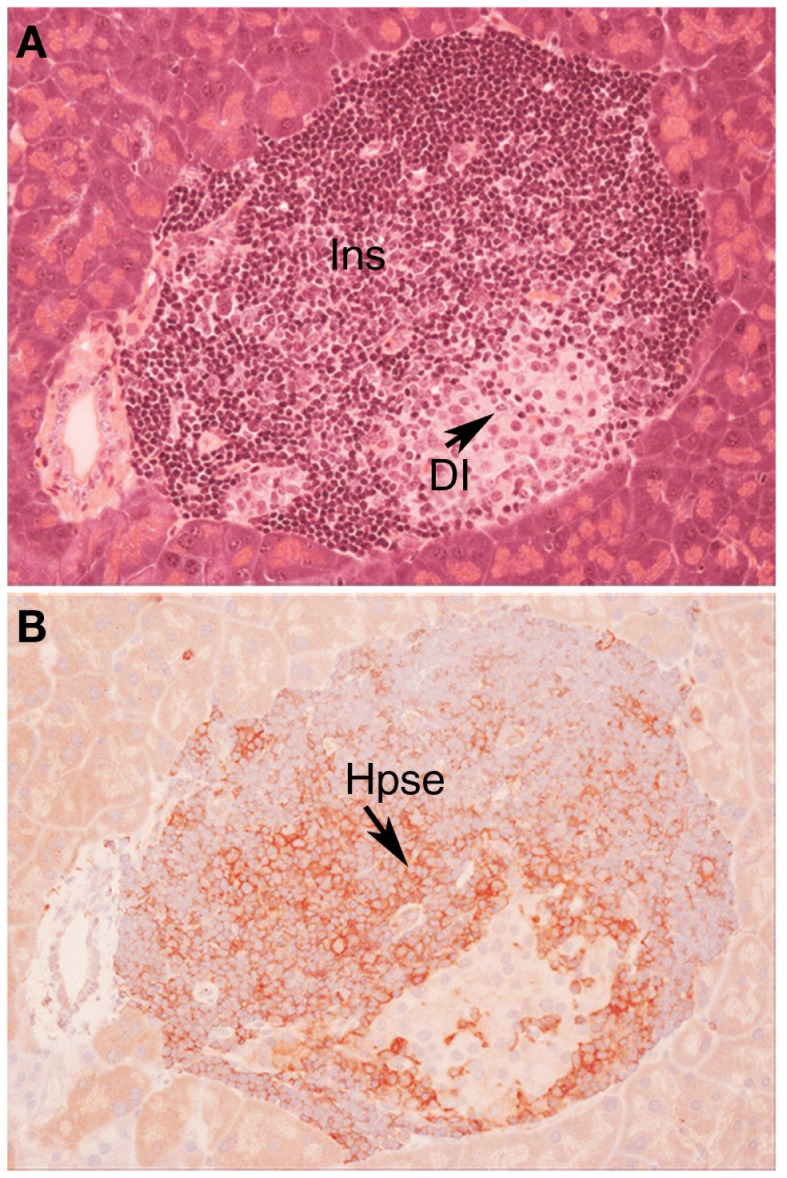
**Autoimmune T1D in NOD/Lt mice is characterized by cell surface expression of heparanase on insulitis leukocytes**. Histology **(A)** and immunohistochemistry **(B)** of a pancreatic islet in a NOD/Lt female mouse at the time of T1D onset shows islet infiltration by destructive heparanase (Hpse)-expressing insulitis leukocytes **(B)** localized particularly at the insulitis-islet interface, a process which leads to loss of beta cell HS and beta cell death. **(A)** hematoxylin and eosin; **(B)** HP130 anti-heparanase mAb. Ins, insulitis mononuclear cells; DI, damaged islet tissue; Hpse, heparanase.

**Figure 2:**
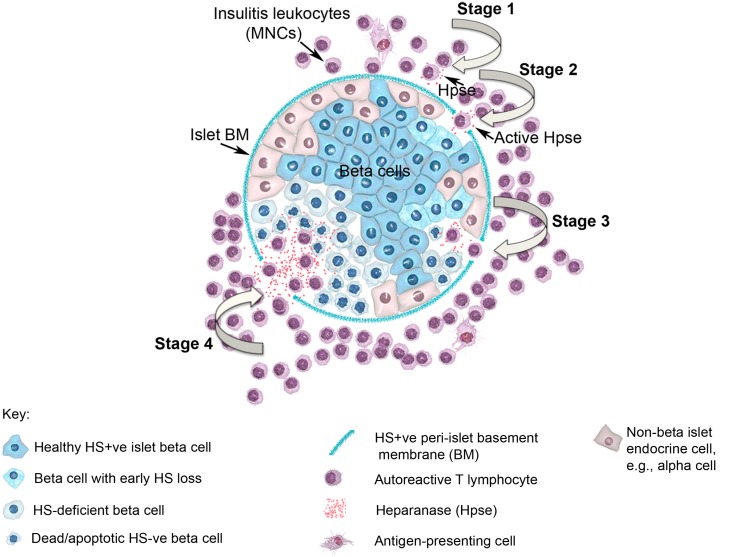
**Diagram showing a pancreatic islet and four stages of T1D disease driven by heparanase and loss of intra-islet HS**. HS (intense blue color) is shown in normal beta cells and in intact peri-islet BM. In Stage 1 of the disease process, non-destructive insulitis mononuclear cells produce heparanase (red dots). Onset of destructive insulitis occurs when heparanase becomes catalytically active and degrades HS in the islet BM (Stage 2). Damage to the islet BM barrier allows activated autoreactive T cells to enter the islet cell mass where the local production of heparanase leads to degradation of intracellular HS in islet beta cells (paling blue color; Stage 3). Progression of HS depletion throughout the islet beta cell population results in increased beta cell death (palest blue color in “shriveled” beta cells; Stage 4), loss of insulin production and ultimately the development of T1D. MNC, mononuclear cell; BM, basement membrane; Hpse, heparanase.

In view of the established role for Hpse in leukocyte migration in other experimental models of inflammation (see above), we predicted that during T1D development, Hpse produced by islet beta cell-specific autoreactive T cells, inflammatory leukocytes, and possibly endothelial cells in the pancreatic vasculature, would be required to degrade HS in the sub-vascular endothelial BM. Thereafter, Hpse-mediated degradation of HS in the underlying pancreatic ECM would allow the inflammatory cells to migrate to individual islets and destroy the islet beta cells. Since T1D development is a chronic disease process, we furthermore speculated that there would be an on-going need for this degradative activity. Our studies identified, however, that the requirement for Hpse extended far beyond the enzymatic activity necessary for leukocyte migration and the establishment of chronic inflammation. Indeed we discovered a critical role for Hpse at the level of the islets themselves. This local involvement of Hpse stemmed from the exceptionally high levels of HS normally associated with the islets *in situ* (8). Initially we confirmed the presence of a BM at the islet periphery (i.e., peri-islet BM) and revealed the HSPG perlecan as a previously unrecognized constituent (47). This HS +ve islet BM was predicted to act as a barrier to invading cells, analogous to the sub-endothelial BM. On further investigation of the distribution of HS in normal mouse islets *in situ*, we found that HS was expressed not only in the islet BM but at extraordinarily high levels throughout the islet cell mass (8). Immunohistochemical studies demonstrated that insulitis mononuclear cells (MNCs) in NOD mice strongly expressed cell surface Hpse (Figure 1; Figure 2, Stage 1). Furthermore, Western blotting analyses showed that the insulitis leukocytes expressed high levels of catalytically active Hpse at the time of diabetes onset in NOD mice, in contrast to the expression of inactive Hpse by peri-islet leukocytes in young pre-diabetic mice (8). Intra-islet infiltration by insulitis MNCs correlated with disruption of the islet BM (Figure 2, Stage 2), loss of the islet BM matrix proteins including the HSPG perlecan (47), progressive loss of intra-islet HS (Figure 2, Stage 3) and beta cell death (Figure 2, Stage 4) (8). Our studies have strongly indicated that such processes are mediated by catalytically active Hpse (Figure 2, Stages 2–4). This newly unveiled role for Hpse has more recently been validated using a transgenic mouse model of acute T1D and adoptively transferred Hpse-knockout effector T cells (unpublished data).

*In vitro* studies of beta cells isolated from normal mouse islets revealed both the unique intracellular localization of HS and its function in maintaining the viability of beta cells (8). Loss of intracellular HS correlated with beta cell death and conversely, the restoration of intracellular HS after culture of the beta cells with HS mimetics, correlated with beta cell survival. HS replacement not only preserved beta cell viability but rendered the beta cells resistant to oxidative damage induced by treatment with hydrogen peroxide [a source of reactive oxygen species (ROS)] (8). Collectively these findings led us to speculate that the intrinsic role of intracellular beta cell HS *in situ* in the pancreas is to protect the beta cells from the physiological levels of ROS generated as a consequence of their high metabolic and biosynthetic activity (8). Furthermore, we reasoned that such a mechanism could compensate for the low levels of free radical scavenger enzymes in beta cells (48).

Together, our *in vivo* and *in vitro* studies identified multiple roles for Hpse in T1D, namely promoting the migration of leukocytes from pancreatic blood vessels (i.e., across the sub-endothelial BM and through the pancreatic ECM), aiding the passage of leukocytes across the islet BM and depleting islet beta cells of the intracellular HS needed for their survival (Figure 2). In support of this new paradigm, *in vivo* treatment of pre-diabetic adult NOD female mice with the Hpse inhibitor/HS mimetic, PI-88, for 180 days significantly delayed T1D onset and reduced the incidence of diabetes by ~50% (8). Compared to saline-treated control NOD mice, PI-88 treatment significantly increased the proportion of pancreatic islets that were intact, significantly reduced the proportion of islets that showed destructive insulitis and better preserved the HS content of the islets (8). This hallmark study has therefore unveiled T1D disease to be largely Hpse-dependent. The extraordinarily high HS content of the beta cells, which is essential for their survival, renders them particularly vulnerable to Hpse-mediated damage. The localization of HS in the islet BM, which by convention acts as a barrier to impede leukocyte infiltration, has also been confirmed in normal human islets (49). Our studies suggest that intracellular HS maintains beta cell survival at least in part, by acting as a “free radical sink,” protecting the beta cells against harmful chemical species generated endogenously. Our findings, which we have subsequently validated in *in vitro* studies of human islets and beta cells (unpublished data), therefore highlight Hpse inhibitors as a new class of therapeutic that can potentially be used to prevent T1D progression in humans.

## Heparanase and Diabetic Complications

The current treatment for T1D is exogenous insulin therapy. While insulin therapy keeps diabetic individuals reasonably healthy, precise control of blood glucose levels invariably fails to be achieved. As a consequence, macrovascular and microvascular diseases develop, resulting in heart disease, nephropathy, retinopathy, and neuropathy. There is compelling evidence that diabetic vascular complications are associated with the accumulation of advanced glycation end products (AGEs) (50). Recent studies have also suggested an important role for Hpse in the development of diabetic nephropathy, a complication pertinent to both Type 1 and Type 2 diabetes. Underpinning this role, involvement of Hpse in proteinuric renal disease has also been established in experimental models of Adriamycin-induced nephropathy and passive Heymann nephritis (22, 51, 52). Diabetic nephropathy is characterized by an increase in the permeability of the glomerular BM (GBM), leading to proteinuria, as well as by tubular and interstitial fibrosis (22, 53). Hyperglycemia has been reported to regulate and, in fact, increase Hpse expression in renal epithelial cells *in vitro* (54). This finding is consistent with the increased expression of Hpse in renal glomeruli (e.g., glomerular podocytes) in human diabetic nephropathy, with significant increased levels of Hpse in the urine of diabetic patients and with a selective decrease in the expression of GBM HS (55–58). Nephropathy resulting from long-term streptozotocin-induced T1D was demonstrated in wildtype mice but not in Hpse-knockout mice (59), supporting a role for Hpse in this condition. The mechanism by which Hpse potentiates diabetic proteinuria may involve altered interactions between glomerular cells and HS-depleted GBM, the release of bioactive molecules from degraded HS, or intracellular signaling in the glomerular cells (22). Hpse is also strongly expressed in renal tubules in diabetic nephropathy and may contribute to tubular fibrosis via effects on FGF-2-signaling (53, 54, 58). An essential role for Hpse in modulating renal tubular morphology was confirmed in diabetic Hpse-knockout mice, which unlike diabetic wildtype controls, were free of histological evidence of tubular fibrosis (59). *In vitro* studies have implicated albumin overload and AGEs, rather than high glucose, in stimulating Hpse expression via the PI3K/Akt pathway in tubular endothelial cells, and in the subsequent loss of cell surface HS (60). Inhibition of both Hpse activity and the expression of tubular fibrosis markers *in vitro* by sulodexide furthermore highlights Hpse as a potential therapeutic target for preventing diabetic renal complications (53, 56).

Heparanase may also play a role in diabetic retinopathy. Increased Hpse expression has been demonstrated in high glucose-treated human retinal endothelial cells and in the retinal vascular endothelium of streptozotocin-induced diabetic rats. *In vitro*, increased levels of active Hpse correlated with enhanced levels of VEGF, a critical angiogenic growth factor required for neovascularization. Upregulation of VEGF in retinal endothelial cells *in vitro* and in the retina of diabetic rats was inhibited by PI-88, supporting a regulatory role for Hpse, possibly by Src activation (61). At the level of leukocyte adhesion to rat retinal endothelium, an early marker of diabetic retinopathy, Hpse inhibition correlated with the decreased expression of the adhesion molecule ICAM-1 as well as VEGF, properties that implicate Hpse in both the arrest of leukocytes in the retinal vasculature and the associated local vascular dysfunction (62). Parallel studies have further demonstrated a more general role for Hpse in high glucose-induced vascular injury and have provided suggestive evidence for a role for Hpse in the pathogenesis of diabetic atherosclerosis (63–65).

## Concluding Remarks

The surprising contribution of Hpse to the pathogenesis of T1D in NOD mice, together with the reported involvement of Hpse in the development of vascular complications of diabetes, highlight the potential application of safe and effective Hpse inhibitors for T1D rescue and treatment. Our studies strongly suggest that therapeutic intervention with dual activity Hpse inhibitors/HS mimetics at early stages of the disease could not only prevent the progression of T1D but potentially also fortify the HS content of any remaining beta cells. By preserving the viability and function of residual insulin-producing beta cells, physiological control of glycemia could be maintained without the need for exogenous insulin therapy. Additional benefits of this therapeutic approach would likely extend to preventing the secondary vascular complications of diabetes. Moreover, the studies reviewed here also support the potential treatment of established T1D with Hpse inhibitors to arrest the progression of diabetic vascular diseases, including nephropathy and retinopathy.

## Conflict of Interest Statement

Each author is a shareholder in the start-up biotechnology company Beta Therapeutics Pty. Ltd., established to evaluate the therapeutic efficacy of dual activity HS mimetics/heparanase inhibitors in preventing the progression of T1D in humans.
